# Draft Genome Sequence of *Bacillus altitudinis* Strain KL4, Isolated from Bottom Sediments in Lake Krotovaya Lyaga (Novosibirsk Region, Russia)

**DOI:** 10.1128/genomeA.01494-17

**Published:** 2018-02-08

**Authors:** Aleksey S. Rozanov, Aleksandra A. Shipova, Alla V. Bryanskaya, L. A. Tekutieva, O. M. Son, Sergey E. Peltek

**Affiliations:** a Federal Research Center Institute of Cytology and Genetics of the Siberian Branch of the RAS, Novosibirsk, Russia; b Far Eastern Federal University, Vladivostok, Russia; c Arnika, Ltd., Vladivostok, Russia

## Abstract

The *Bacillus altitudinis* strain KL4 was isolated from bottom sediments in Lake Krotovaya Lyaga (Novosibirsk Region, Russia, 53.7°N, 77.9°E). The sequenced and annotated genome is 3,738,419 bp long and carries 3,909 genes.

## GENOME ANNOUNCEMENT

The species *Bacillus altitudinis* is a microorganism found in air samples (collected at an altitude of 41 km) and was first described in 2006 (1) and later isolated from other environments, including the Uzon caldera. This bacterium produces RNase, which has antitumor and antiviral properties (2). *B. altitudinis* can be used as an efficient microorganism for the enhancement of plant growth and suppression of fungal disease (3).

The *Bacillus altitudinis* strain KL4 was isolated from bottom sediments in Lake Krotovaya Lyaga (Novosibirsk Region, Russia, 53.7°N, 77.9°E). We isolated the strain for collection of biotechnological microorganisms as a source of novel promising subjects for biotechnology and bioengineering for the Federal Research Center Institute of Cytology and Genetics of the Siberian Branch of the RAS.

A *Bacillus altitudinis* strain KL4 culture was cultivated in liquid medium containing 1% trypton, 0.5% yeast extract, and 1% of NaCl. Eight milliliters of cell culture was pelleted by centrifugation and resuspended in 75 µl H_2_O by intense pipetting. DNA was isolated using a DNA purification kit (Fermentas). A NEBnext Ultra II DNA library prep kit for Illumina (New England Biolabs, USA) was used to create libraries for genome sequencing. Genome sequencing was performed on a MiSeq system (Illumina), using a MiSeq reagent kit v3 for 150 cycles (Illumina, USA) in the Molecular and Cellular Biology facility at IMCB SB RAS.

*De novo* assembly of short reads into contigs was performed using SPAdes v. 3.10.1 (4). Contigs shorter than 1,000 bp were deleted. A total of 35 contigs yielded a genome sequence 3,738,419 bp long, and the GC content is 41.36%. Open reading frame (ORF) prediction and automatic annotation were performed using NCBI PGAAP (https://www.ncbi.nlm.nih.gov/genome/annotation_prok). The complete genome sequence contained 3,909 genes, 3,790 coding sequences (CDS), rRNAs (1 5S, 5 16S, and 1 23S), 60 tRNAs, and 5 noncoding RNAs (ncRNAs).

### Accession number(s).

The draft genome sequence for *Bacillus altitudinis* strain KL4 has been deposited in DDBJ/EMBL/GenBank under the accession no. PEKO00000000. The 35 contigs have been deposited under accession no. PEKO02000001 to PEKO02000035.
